# Energetic Constraints on Species Coexistence in Birds

**DOI:** 10.1371/journal.pbio.1002407

**Published:** 2016-03-14

**Authors:** Alexander L. Pigot, Joseph A. Tobias, Walter Jetz

**Affiliations:** 1 Groningen Institute for Evolutionary Life Sciences, University of Groningen, Groningen, The Netherlands; 2 Edward Grey Institute, Department of Zoology, University of Oxford, Oxford, United Kingdom; 3 Department of Life Sciences, Imperial College London, Silwood Park, Ascot, United Kingdom; 4 Department of Ecology and Evolutionary Biology, Yale University, New Haven, Connecticut, United States of America; Ecole Normale Supérieure, FRANCE

## Abstract

The association between species richness and ecosystem energy availability is one of the major geographic trends in biodiversity. It is often explained in terms of energetic constraints, such that coexistence among competing species is limited in low productivity environments. However, it has proven challenging to reject alternative views, including the null hypothesis that species richness has simply had more time to accumulate in productive regions, and thus the role of energetic constraints in limiting coexistence remains largely unknown. We use the phylogenetic relationships and geographic ranges of sister species (pairs of lineages who are each other’s closest extant relatives) to examine the association between energy availability and coexistence across an entire vertebrate class (Aves). We show that the incidence of coexistence among sister species increases with overall species richness and is elevated in more productive ecosystems, even when accounting for differences in the evolutionary time available for coexistence to occur. Our results indicate that energy availability promotes species coexistence in closely related lineages, providing a key step toward a more mechanistic understanding of the productivity–richness relationship underlying global gradients in biodiversity.

## Abstract 2

An analysis of phylogenetic and geographic data across the world's bird species reveals that the probability of species coexistence is constrained by the availability of energy in the environment.

## Author Summary

The increase in the number of species with the availability of energy in the environment is one of the most general but least understood patterns in global biodiversity. The finite amount of energy flowing through an ecosystem has long been suspected to place a fundamental constraint on the ability of species to subdivide ecological resources, with greater energy availability—and, thus, ecosystem productivity—in the tropics, potentially facilitating increased levels of coexistence. However, empirical support for this hypothesis has been lacking, raising the possibility that richness is higher in productive ecosystems for largely historical reasons, including the greater geological age and area of tropical biomes, which increases the period of time available for diversity to accumulate. By combining phylogenetic and geographic data from across the world’s bird species, we show that greater ecosystem productivity is associated with an increased probability of coexistence among closely related lineages and that this pattern contributes to the higher species richness in the tropics. Our results confirm that contemporary gradients in species richness are fundamentally shaped by energetic constraints on coexistence.

## Introduction

The relationship between species richness and energy availability—often described in terms of ecosystem productivity—is widespread yet poorly understood [1–5]. A link between the flux of energy through an ecosystem and the number of species it contains has long been recognised [6], but the exact form of the relationship and its scale-dependence have traditionally been the focus of much debate [4,7–11]. Recent analyses have established that, when measured over large geographic and taxonomic scales (>50 km grain size across continental or global study regions), species richness increases strongly with the availability of potential energy (e.g., net primary productivity or associated climatic proxies [12]), and that this relationship explains much of the spatial variation in biodiversity [13–17]. Similar patterns are repeated across a variety of life forms and regions with contrasting evolutionary histories, implying that energy availability may offer a universal explanation for Earth’s major gradients in biodiversity [1,18]. However, resolving the processes driving this relationship has proven far more challenging [2,3,19,20].

The predominant explanation for the positive relationship between energy availability and species richness (the energy–richness relationship) is that the amount of energy flowing through an ecosystem places a fundamental constraint on the number of species coexisting at any single point in space (alpha-diversity) [2,3,6]. Higher energy fluxes and the corresponding expansion in the breadth and availability of useable resources are expected to reduce the incidence of stochastic population extinction by sustaining a larger total number of individual organisms (the “more individuals” hypothesis) [18,21] and to increase the potential for local niche partitioning required for stable coexistence [22–24]. However, direct support for these hypotheses is very scant [2], suggesting that the energy-richness relationship may have arisen through an alternative process [19,25]. For instance, species may have diversified more rapidly in productive environments because higher temperatures [26], increased solar radiation [27], larger population sizes [21], or reduced dispersal ability [28] drive accelerated rates of speciation (the “speciation rates” hypothesis) [2,29–31]. Alternatively, if most clades originated in the humid tropics, then species richness may simply have had a longer time to accumulate in regions with high energy availability, while strongly conserved physiological constraints have prevented the expansion of species or clades into colder or drier environments with lower productivity (the “niche conservatism” hypothesis) [11,14,25,32–34]. Thus, rather than reflecting energetic constraints on community assembly, more species may coexist in productive ecosystems for largely historical reasons [2,35].

These contrasting historical explanations have not been ruled out by previous studies because standard methods for testing the relationship between energy availability and limits to species coexistence are largely indirect, including correlations between energy availability and assemblage biomass [36,37], population density [24,38,39], and rates of local extinction [40]. While these correlations are suggestive of a link between energy availability and community assembly dynamics, they do not conclusively establish whether coexistence is subject to energetic constraints, nor whether such constraints drive broad-scale gradients in species richness [2,3]. An alternative approach has been to examine the relationship between energy availability and the richness of entire assemblages, while statistically accounting for differences in regional diversity [14,19,41–44]. However, this method is problematic because it lumps together numerous unrelated species spanning a vast array of lifestyles and diets, with only minor ecological overlap, and ignores the possibility that regional species diversification may itself ultimately be regulated by local limits to coexistence [45,46]. Understanding the role of energetic constraints on biodiversity therefore requires an approach that focuses on species with the broadest overlap in ecological niches, while robustly accounting for processes playing out over evolutionary time.

Here, we address these issues using a comparative framework to explore patterns of geographic range overlap among sister species, relatively young lineages for which energetic constraints on coexistence resulting from similarity in resource use are expected to be most pronounced [47,48]. Our analyses include data from 1,021 sister pairs, distributed across the avian phylogenetic tree and the world’s major landmasses, and representing 30% of all species for which genetic sequence data are available (S1 Fig) [49]. The breadth of this dataset and the evolutionary context provided by the phylogenetic relationships among lineages allows us to test the extent to which energy availability explains species coexistence in birds as well as the relationship between coexistence and contemporary gradients in species richness.

We first quantify patterns of coexistence on the basis of overlap in the breeding distributions of sister species and test whether energy availability explains the probability of coexistence, both across species pairs and geographic space. Our analysis accounts for the potentially confounding effects of other abiotic variables and the phylogenetic nonindependence of species. We then take advantage of the fact that almost all speciation events in birds have involved a phase of geographic isolation [46,50], using estimated divergence times to test how ecosystem productivity regulates the temporal dynamics of coexistence following divergence in allopatry [47,51]. We compare a model in which energy availability predicts either the initial rate at which coexistence is attained, or its temporal duration, to a null model in which variation in the incidence of coexistence is explained purely by differences in the time elapsed since speciation [47]. Throughout, we account for uncertainty in phylogenetic relationships and estimates of divergence times by fitting our models across multiple trees sampled at random from the posterior distribution [49]. In a final set of analyses, we assess the relationship between patterns of species coexistence and the total richness of avian assemblages using the geographic distributions of all 9,993 bird species. By combining these approaches, we aim to clarify the role of energetic constraints in both the establishment and maintenance of species coexistence as well as the contribution of these dynamics to global-scale gradients in species richness.

## Results and Discussion

### The Geography of Species Coexistence

**Fig 1:**
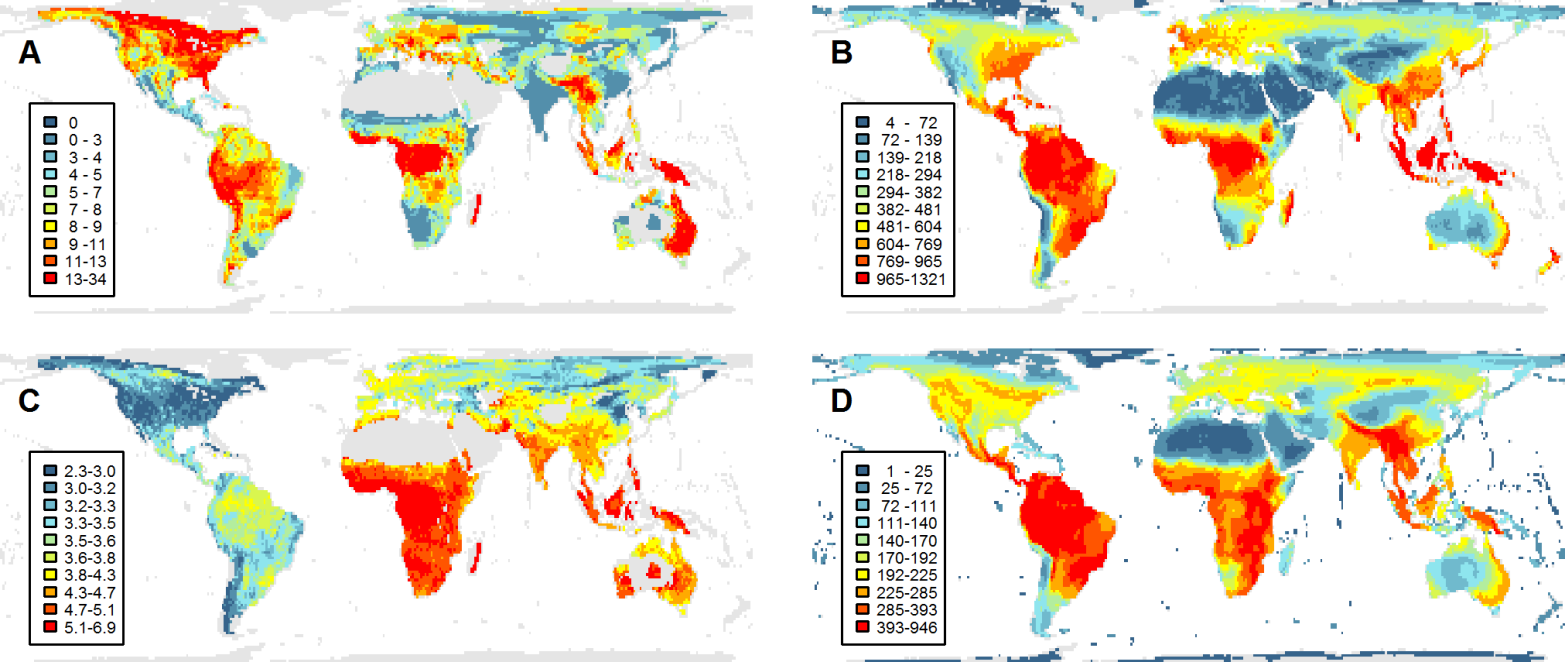
Observed geographic variation in (A) the incidence of coexistence of sister species *(n* = 1,021 pairs), (B) energy availability measured as net primary productivity (NPP; g C m^-2^ yr^-1^), (C) the median age of sister species (million years [Ma]), and (D) the total assemblage richness of all birds (*n* = 9,993). In (A), colours denote the percent of pairs within a quadrat that coexist locally (i.e., both present within that quadrat). Analysis grain size is 110 km, colours follow quantile classification (i.e., equal number of cells in each class), and, in (A,C), only quadrats containing at least 20 sister pairs are plotted. See Dryad depository for cell values [52].

Across our dataset, 28% of sister species coexist, with the rest having either geographically isolated distributions or exhibiting only marginal overlap along narrow contact zones (area of range overlap <20% of the smaller species range; see Materials and Methods). To examine the incidence of local coexistence and how this varies across geographic space, we quantified the percentage of sister species pairs coexisting within equal area quadrats (resolution of 110 km x 110 km, ≈ 1° at the equator). Few coexisting sister species are sympatric over the entirety of their geographic range (mean range overlap = 66% of the smaller species range and 22% of the total geographic range of both sister species combined), resulting in a low average incidence of coexistence among sister species at the local scale (mean percent of sister pairs in a cell where both species are locally present = 7%). However, levels of local coexistence exhibit substantial variation across geographic space (0%–34%; Fig 1A). Areas containing a particularly high concentration of coexisting lineages occur throughout the wet tropics, including the eastern slope of the Andes and Amazonia, the Congo basin, New Guinea, the eastern Himalayas, and the Malay Archipelago. Beyond the tropics, additional regions of high coexistence occur along the eastern coast of Australia and throughout the northern Nearctic (Fig 1A).

### Energy Availability and the Incidence of Species Coexistence

**Fig 2:**
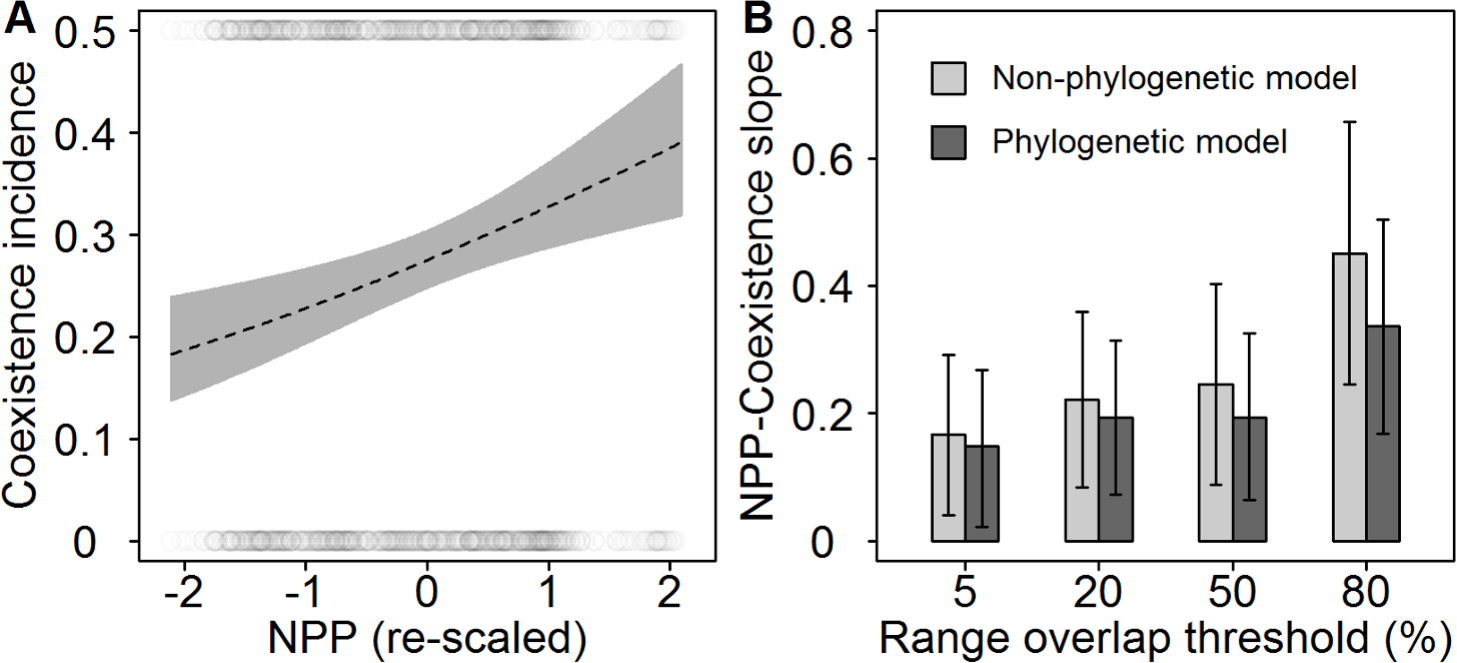
The relationship between (A) coexistence and energy availability (NPP) across sister species pairs is positive and (B) strengthens with the area over which species coexist (as a percentage of the smaller species range). In (A), the dotted black line (and grey 95% confidence interval bands) indicates the predicted probability of coexistence as a function of NPP (standardised to unit variance) and using a threshold of 20% range overlap to define coexistence (S1 Table). Grey circles indicate the observed distribution of sympatric and allopatric sister pairs across the global NPP gradient (N.B. because of the low overall incidence of coexistence, the *y*-axis is plotted from 0 to 0.5). In (B), bars show estimated slope parameters (and 95% confidence intervals) from a generalised linear model ignoring (light grey) or accounting for (dark grey) species phylogenetic covariance under different coexistence thresholds (S1 Table). See Dryad depository for sister species pairs and plotted data [52].

We used sister species pairs to assess patterns of coexistence, thus avoiding the pseudoreplication introduced when analysing assemblage level patterns. The energy available for each sister pair was quantified by averaging mean annual net primary productivity (NPP; Fig 1B) across their combined geographic distribution (for allopatric pairs) or those grid cells where both species coexist (for sympatric pairs) (Materials and Methods). We found that the probability of coexistence between sister species increases strongly with local NPP (generalised linear model [GLM], slope = 0.22, *p* < 0.01; Fig 2A). This significant positive association was evident regardless of the degree of geographic range overlap used to define coexistence (5% to 80%), but became increasingly steep when considering more stringent overlap thresholds (Fig 2B).

NPP is the most appropriate metric for testing energetic constraints in heterotrophic organisms, but we also detected the same significant trend using actual evapotranspiration (AET) to quantify potential energy (GLM, slope = 0.15, *p* < 0.05). In both cases, the effect of energy availability on coexistence was nonlinear, with the inclusion of a positive quadratic term leading to a substantial improvement in model fit (GLM, slope = 0.21, quadratic = 0.21, *p* < 0.01, Δ Akaike information criterion [AIC] = 8.2 in favour of a model including a quadratic term). Thus, while it has long been debated whether increased ecosystem productivity may elevate the intensity of competition, thereby reducing coexistence under conditions of high resource availability [4,22], we find the opposite pattern, wherein the probability of coexistence is a positive accelerating function of energy availability.

If the incidence of coexistence between sister species shows a strong phylogenetic signal, then the positive effects of energy availability could be driven by one or a few individual clades. To evaluate this possibility, we calculated the D statistic, which provides an estimate of the phylogenetic signal in a binary character relative to both a phylogenetically random distribution (expected D = 1) and a Brownian motion model of trait evolution (expected D = 0) [53]. We found that while the incidence of coexistence was not randomly distributed across the avian tree (*p*_[D = 1]_ < 0.01), phylogenetic signal was low (D = 0.88), indicating that any tendency for coexistence to be clustered in particular clades is weak (Materials and Methods; S1 Fig). Furthermore, when we included the phylogenetic covariance between species as a random effect in our models, the significant positive association between energy availability and coexistence remained (Fig 2B and S4 Table).

Another possibility is that the relationship between species coexistence and energy availability could arise due to covariation with other environmental factors. For instance, it has been proposed that resource specialisation leading to coexistence may be precluded in more seasonal environments in which resource abundance undergoes larger short-term temporal fluctuations [2]. Fluctuations in climate and resources may also play a role over longer time-frames, and it is variously predicted that coexistence could be promoted [54] or reduced [55] in regions covered by ice sheets during recent glacial maxima. Other physical attributes of the environment are thought to promote coexistence, including topographical heterogeneity [56] and ambient temperature [57]. To address these possibilities, we assessed the role of energy availability on coexistence relative to a suite of abiotic variables in both single and multipredictor models (all variables were normalised to allow effect sizes to be directly compared).

**Fig 3:**
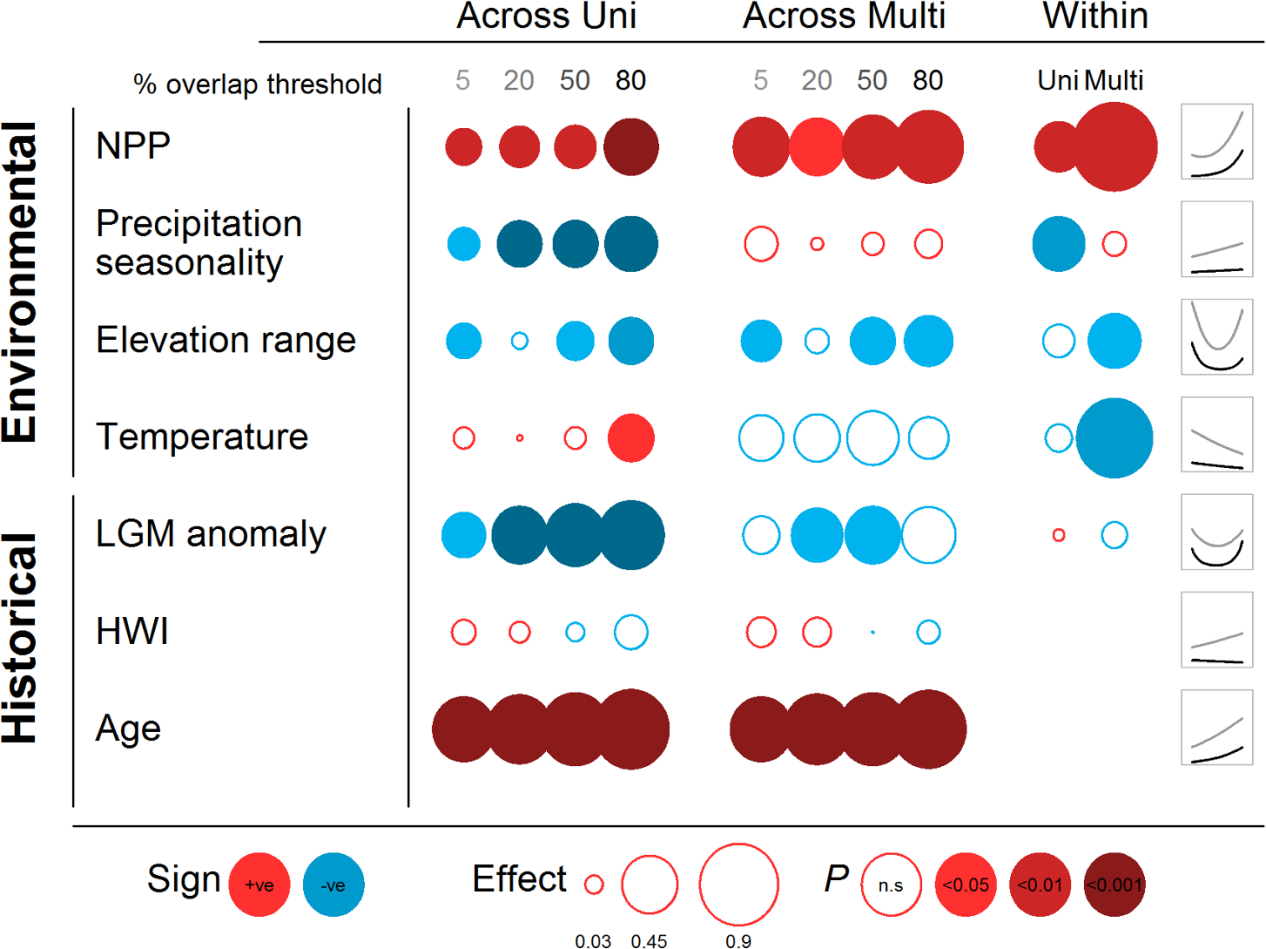
Environmental and historical predictors of species coexistence. Symbol sizes and colour denote estimated linear slopes, direction, and significance of each variable in both univariate (“Uni”) and multivariate (“Multi”) generalised linear models. Results are shown for different coexistence thresholds (5%–80% range overlap) and for models predicting coexistence across *(n* = 1,021 pairs) and within *(n* = 187 pairs) sister species pairs. Icons denote the relationship shape for each variable in the across-pair multivariate model assuming either a low (5%, light grey) or conservative (80%, dark grey) overlap threshold. For quadratic parameter estimates, see S1 Table. HWI is the “hand-wing index,” a measure of wing shape and a proxy for dispersal ability in birds (Materials and Methods). See Dryad depository for plotted data [52].

In addition to the effects of energy availability, our models highlighted a number of significant predictors of species coexistence (Fig 3, S1–S4 Tables). When assessed in isolation, we found that coexistence was significantly reduced in areas experiencing greater environmental seasonality. However, seasonality was not significant in a multipredictor model, suggesting that these effects arise from covariation with other abiotic predictors. We also detected negative relationships between coexistence and both the change in temperature since the last glacial maximum and topographic heterogeneity. In both cases, the relationship is distinctly U-shaped, with coexistence first declining and then increasing. These inconsistent slopes suggest that neither long-term climatic variability nor topographic heterogeneity have a general or direct mechanistic effect on coexistence. In particular, our results suggest that the effect of topographic heterogeneity may be sensitive to the inaccuracies in broad scale distribution maps in mountainous regions [58]. This is because, when we used more stringent overlap thresholds to define coexistence, the slope of the relationship between topographic heterogeneity and coexistence shifted to become increasingly negative and monotonic (Fig 3, S1–S3 Tables).

Finally, contrary to the well-established positive relationship between species richness and temperature in birds [59,60], we found that ambient temperature was unrelated to the probability of coexistence. This finding suggests that temperature may be associated with richness for historical reasons (i.e., tropical niche conservatism) rather than because of any direct mechanistic link with the maintenance of species diversity. Importantly, we found that when we statistically accounted for all these additional abiotic variables, the positive effect of NPP on coexistence was strengthened (Fig 3, S1–S4 Tables).

### Energy Availability and the Dynamics of Species Coexistence

Because the vast majority of speciation events in birds are thought to require a period of geographic isolation, we can assume that coexistence between species only arises at a later stage following the expansion of species geographic ranges [61]. The median age of sister species varies geographically, being highest in Australasia and the Old World tropics and decreasing toward both high northern latitudes and the New World (Fig 1C). Because of variation in the time available for range expansions [47,62,63], these gradients in sister species age may contribute to geographical differences in the incidence of coexistence and its environmental correlates. Furthermore, rates of geographic range expansion may vary across species due to differences in intrinsic dispersal ability. For instance, recent evidence from New World birds [51] reveals that the rate at which sympatry is attained increases with species hand-wing index (HWI), a measure of wing-shape correlated strongly with long-distance flight ability [64]. Robustly establishing the role of energy availability in limiting coexistence thus requires accounting for these effects.

To address this, we modelled the probability of coexistence as a function of both species age and HWI in isolation and alongside energy availability and other abiotic predictors (Materials and Methods). As expected, the probability of coexistence increases strongly with species age because sympatric sister pairs are, on average, >1 million years older (4.68 million years [Ma]) than allopatric pairs (3.51 Ma) (Fig 3). Having accounted for both species phylogenetic relatedness and abiotic factors, we found evidence that coexistence is promoted by high dispersal ability (S4 Table). However, this effect was relatively weak and was not significant in our standard nonphylogenetic analysis (Fig 3, S1–S3 Tables). Overall, while our analysis highlights the important contribution of dispersal to current patterns of coexistence, this is unlikely to explain our results, because energy availability retained its independent effects even after accounting for these historical factors (Fig 3, S1–S4 Tables). We note that all these results were robust to phylogenetic uncertainty in sister species relationships and divergence times and held regardless of whether we fitted our model across either the Bayesian posterior distribution of trees or the single most credible tree (S1–S4 Tables).

To further explore the effects of energy availability, we extended our comparative framework to test whether energy also predicts the geographical patterns of coexistence within species pairs (S3 Fig; Materials and Methods). This test is more conservative as it controls for differences in time since divergence [65,66], species traits (e.g., dispersal ability), potential geographic variation in taxonomic practices and species description, and factors that may co-vary with energy availability but that are not easily accounted for in comparisons across species pairs (e.g., variation in rates of ecological divergence) [47]. Within-species pair analyses confirmed that energy availability is higher in grid cells where both sister species coexist than in cells where only one sister is present (GLM, slope = 0.31, quadratic = 0.33, *p* < 0.01, *n* = 187, S1 Table), a result that was further strengthened when we statistically accounted for other abiotic variables (Fig 3 and S1 Table). These results strongly reject the possibility that patterns of coexistence arise simply due to historical factors, including differences in species age or rates of niche evolution.

Looking beyond deterministic associations, it is also important to consider whether our results may be explained by stochastic effects. Although highly controversial [17,67], it has been argued that broad-scale gradients in both species richness and its environmental associations may be driven by random range dynamics within a bounded geographic domain, the so-called “mid-domain effect” [68,69]. However, our findings are inconsistent with this stochastic model because we show that current energy availability predicts not only the broad-scale variation in range overlap, but also the particular locations of coexistence and allopatry within sister pairs (Fig 3 and S1 Table). These results confirm previous evidence demonstrating that random geographic range expansion cannot explain species distributions of birds [70].

When viewed from a historical perspective, the positive relationship between coexistence and energy availability may be generated either because more productive ecosystems facilitate the initial transition to sympatry following speciation [46,71], or because they prolong the duration of coexistence by reducing rates of local extinction [40]. To examine these possible mechanisms, we applied a stochastic approach to model the dynamics of coexistence between species over evolutionary time (Materials and Methods) [47]. In this model, we assumed that sister species are spatially isolated at the time of population divergence and then transition to a state of coexistence at a constant rate, σ. Because local extinction may result in coexisting species returning to a state of spatial segregation, we incorporate this process in our model by allowing reverse transitions back to allopatry at rate ε. Based on this model, we obtained the likelihood of observing sister species pairs in their current geographic state (allopatric/parapatric or sympatric) given the empirical distribution of sister species ages. We then used likelihood optimisation to estimate the transition rates to (σ) and from (ε) coexistence, from which the expected waiting time to sympatry following speciation (1/σ) and the subsequent expected duration of coexistence (1/ε) can be calculated (Materials and Methods). By comparing AIC scores, we evaluated the relative fit of an “energy-availability dependent” (EAD) model, in which either σ, ε, or both are allowed to vary as a log-linear function of NPP, to a null model, in which transition rates between geographic states are equivalent across species. Using this approach, we were able to provide estimates of coexistence dynamics that are independent of any geographic gradient in sister species ages (S1 Text and S4 Fig).

According to our transition model, the mean expected waiting time to coexistence (i.e., 1/σ) following speciation is 5.56 Ma, confirming our previous results highlighting the importance of time in the build-up of species coexistence (S5 Table). Furthermore, in accordance with our standard statistical models, we found that an EAD model fits the data best, rejecting the null hypothesis that the probability of coexistence depends only on sister species age and, thus, the time available for range expansion (S5 Table). A model in which ε decreases with ecosystem productivity (ΔAIC = 7.92 in favour of the EAD model) outperforms a model in which productivity influences σ (ΔAIC = 5.72 relative to the null model), and there was no further improvement in model fit when combining the effects of energy availability on both σ and ε (ΔAIC = 6.05 relative to the null model; S5 Table). The effects of energy availability on ε inferred by our model are substantial, with the mean expected duration of coexistence (i.e., 1/ε) being two times longer in high (4.15 Ma; first NPP quartile) compared to low energy environments (2.04 Ma; fourth NPP quartile).

**Fig 4:**
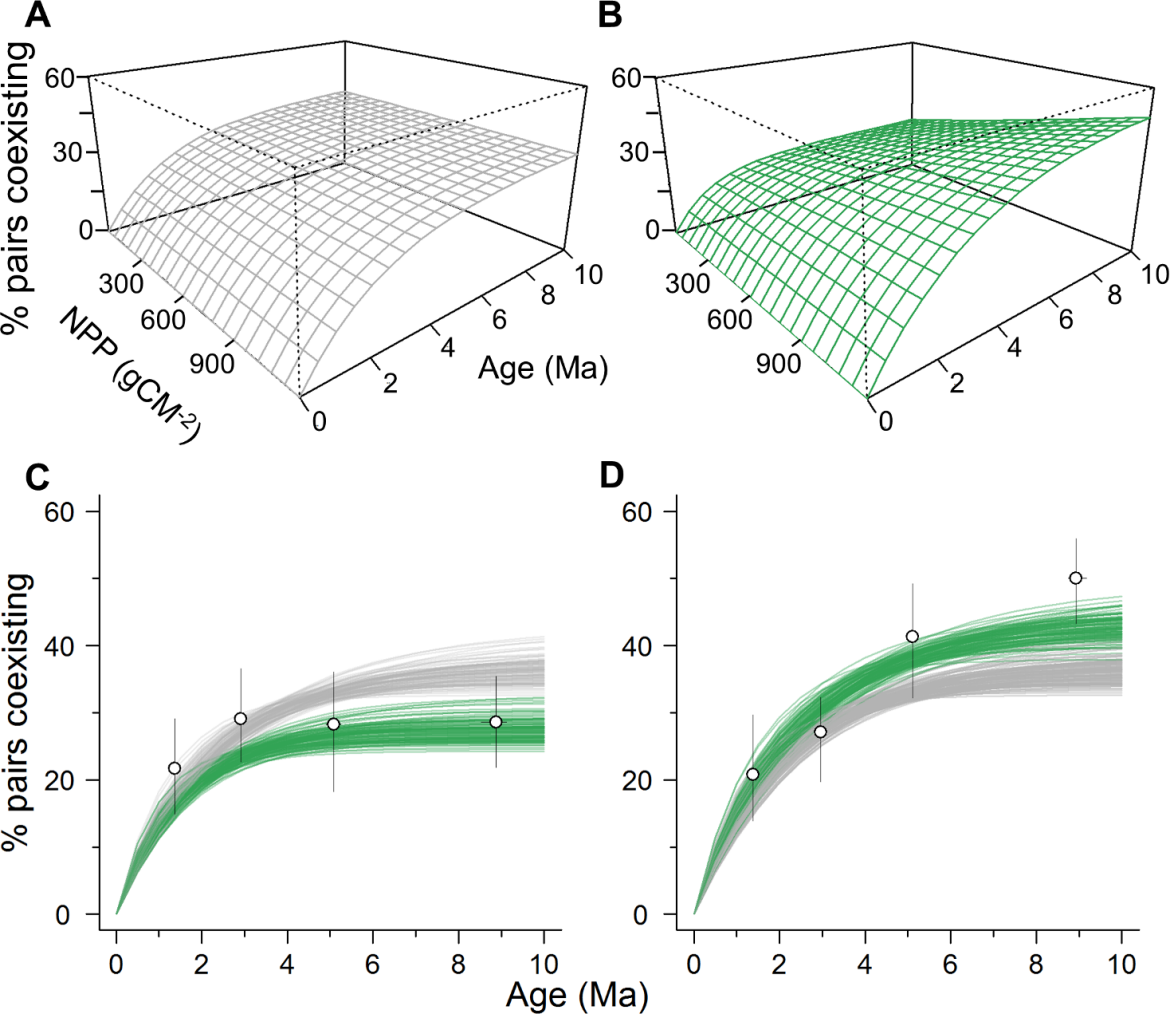
The dynamics of coexistence across the global NPP gradient. (A,B) Expected incidence of coexistence as a function of time since speciation according to (A) the null model in which transition rates to (σ) and from (ε) coexistence are independent of NPP, and (B) the best-fit “energy-availability dependent” (EAD) model in which ε decreases with NPP. (C,D) The match between the expected (lines) and observed (points) incidence of coexistence for lineages in ecosystems with (C) low energy availability (first NPP quartile, <427 gCM^-2^ yr) and (D) high energy availability (fourth NPP quartile, >968 gCM^-2^ yr). Coloured lines highlight variation in expected trajectory across the posterior distribution of trees according to the null (grey) and EAD (green) model. Observed data are for illustration only and indicate the median (and 95% confidence interval, black lines) incidence of coexistence and species age within age quartiles across the posterior distribution of trees. To aid visualization, for plotting maximum sister species age was set to 10 Ma. See Dryad depository for plotted data [52].

To examine whether these inferred coexistence dynamics are consistent with the patterns observed across sister species, we plotted how the probability of coexistence predicted by our model increases as a function of both age and NPP (Fig 4A and 4B). In contrast to the poor fit of the null model (Fig 4A), we find that an EAD model accounting for the effects of productivity on coexistence duration (Fig 4B) better captures the observed variation in the incidence of coexistence across the global gradient in both species age and energy availability (Fig 4C and 4D; Materials and Methods). In particular, this model explains both the similar levels of coexistence observed among recently diverged species, regardless of local energy availability, and the apparent increase in the effect of energy availability over time as differences in the duration of coexistence are realised. Thus, our results suggest that the primary effect of energy availability is not brought about by increasing the rate at which coexistence is attained following speciation, but rather by extending the duration of coexistence, thus allowing a greater accumulation of sympatric diversity.

Recent evidence based on the age of sympatric sister lineages of New World birds suggested that sympatry is attained more rapidly at high latitudes compared to the tropics [54]. This pattern has been explained in terms of the large-scale shifts in habitats following the retreat of Northern Hemisphere ice sheets, with vacant ecological niche space facilitating geographic range expansions [54]. Given the general decline in ecosystem productivity away from the equator (Fig 1B), such a latitudinal increase in rates of secondary sympatry would appear to be at odds with the strong positive effect of energy availability on coexistence reported here. To resolve this, we used our analytical approach to fit a “latitudinal dependent” (LD) model and examined how the dynamics of coexistence varies with absolute geographic latitude. We fitted this model both globally (*n* = 1,021 sister pairs) and within the New World (i.e., the Nearctic and Neotropical realms described by Olson et al. [72]; *n* = 492 sister pairs). Our results reaffirmed a positive effect of latitude on the transition rate to sympatry in New World birds (ΔAIC = 2.19 in favour of the LD model; S6 Table), likely contributing to the high levels of coexistence found across the northern Nearctic (Fig 1A). However, when we extended our analysis globally we found no effect of latitude on the dynamics of coexistence (ΔAIC = 1.63 in favour of the null model; S6 Table). Our results thus indicate that a latitudinal gradient in the rate of secondary sympatry is not a general trend but only a regional phenomenon, and that this does not override the positive effect of energy availability on the duration of coexistence at global scales.

### Coexistence and Species Richness

By demonstrating that high energy availability enhances species coexistence, our results provide a long-sought mechanistic link between current environmental conditions and broad-scale gradients in species richness. However, sister species pairs typically comprise only a fraction of all species within an assemblage, and the extent to which energetic constraints on coexistence contribute to variation in species richness remains unclear. To explore this, we examined how the incidence of coexistence across grid cells is related to the assemblage richness of the 2,042 sister species analysed and to the total richness of all 9,993 bird species (Fig 1D).

**Fig 5:**
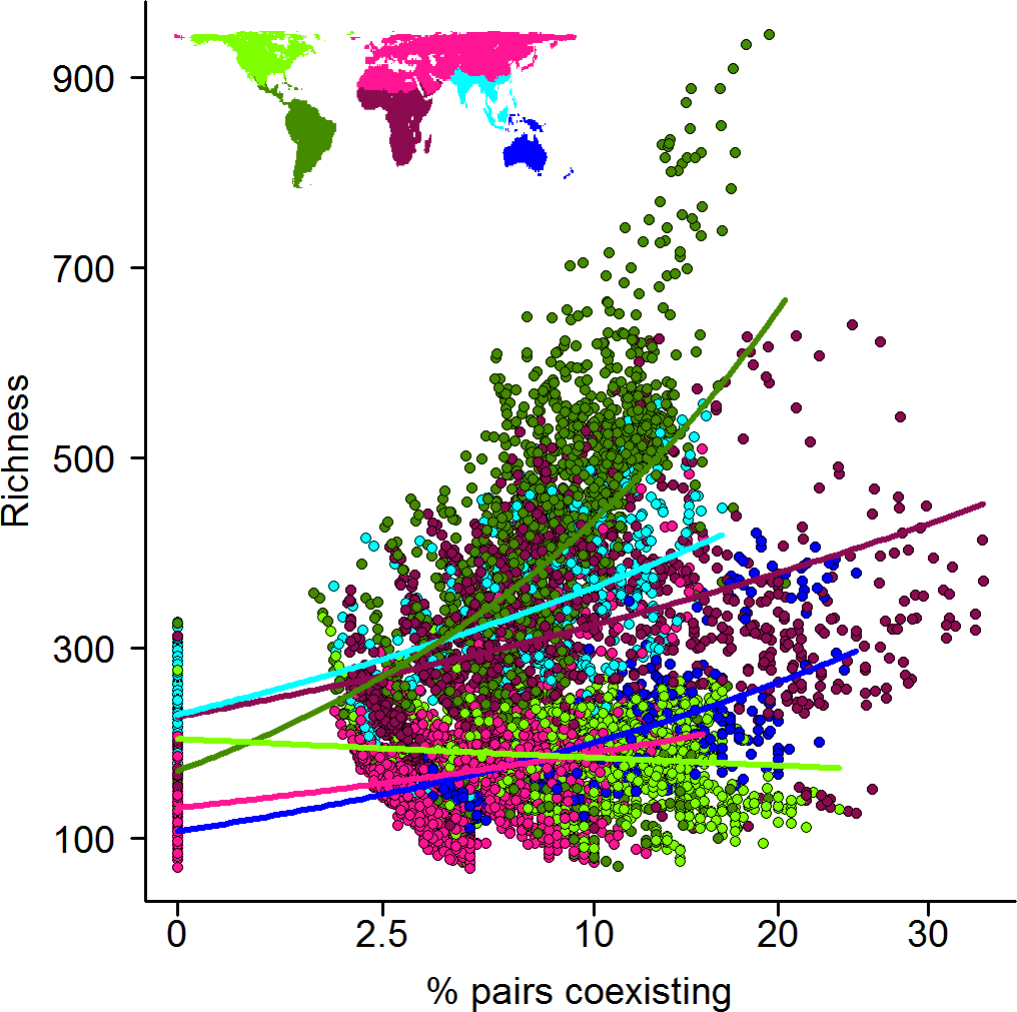
The relationship between the incidence of sister coexistence (%) (*n* = 1,021 pairs) and the total assemblage richness of all birds (*n* = 9,993 species). Slopes are from a generalised linear model (GLM) with quasi-Poisson error structure showing the interaction between “Realm” and “Coexistence” (S7 Table). Only cells containing at least 20 sister pairs were used. Line and point colours denote Realm; see map inset. See Dryad depository for cell values [52].

The results of these approaches revealed that, for a given level of coexistence, species richness is highly variable. Thus, we found a positive but relatively weak association between coexistence and both sister species richness (Spearman’s ρ = 0.36, *p* <0.001) and total avian richness (Spearman’s ρ = 0.34, *p* <0.001) (S2 Fig). However, the relationship between richness and coexistence is triangular with a clear upper boundary, so that maximum species richness increases strongly with the percentage of coexisting sister species (Fig 5). In other words, while high levels of coexistence can be found regardless of local richness, species-rich locations are uniquely those supporting high levels of coexistence rather than simply a large number of allopatric lineages (Figs 5 and S2).

To examine this relationship in more detail, we divided the earth’s land surface into six biogeographic realms [72], each of which has a largely independent evolutionary history and contrasting average levels of species richness (Fig 5). We found that the positive relationship between coexistence and richness was replicated within realms. Nonetheless, there was also evidence of significant interrealm variation in both model slopes and intercepts, potentially reflecting historically driven differences in the richness of regional species pools (Fig 5 and S7 Table) [14]. Having accounted for this between-region variation, correlations between coexistence and richness were substantially strengthened compared to the global model (Afrotropics: ρ = 0.54, Australasia: ρ = 0.80, Indomalaysia: ρ = 0.72, Neotropics: ρ = 0.53, Palearctic: ρ = 0.34, *p* < 0.001 in all cases). The sole exception to this pattern was the Nearctic, where coexistence was negatively correlated with richness (ρ = -0.11). The consistent positive effect of sister species coexistence on assemblage richness is at odds with purely historical explanations for richness gradients based solely on differences in opportunities for species diversification [11,29] and also challenges the idea that high levels of coexistence among sister species simply reflects a lack of community saturation in more depauperate biotas [54,73]. Instead, our analysis confirms the significant role of range expansions in establishing broad-scale gradients in species richness.

### Integrating Macroecological and Macroevolutionary Perspectives to Understand Species Richness Gradients

A robust demonstration of the fundamental relationships linking energy availability, coexistence, and assemblage richness has hitherto been lacking because of the difficulties in accounting for purely historical processes, including variation in the size of regional species pools or differences in the evolutionary time available for speciation and range expansion [14,25,74]. By focusing on interactions between avian sister species of known evolutionary age, we have shown that the probability of coexistence increases with energy availability and that this effect cannot be explained by such historical artefacts. We further demonstrate that the geographical variation in levels of coexistence among closely related lineages is strongly aligned with observed gradients in assemblage richness, supporting a mechanistic link between the global-scale increase in species richness with energy availability.

The increasing application of molecular phylogenetic data to understanding macroecological patterns has often highlighted the importance of evolutionary history in the origin of broad-scale richness gradients, with many studies supporting a model in which the increase in richness with energy availability arises largely as a byproduct of accelerated rates of species diversification or the greater age and area of tropical biomes [11,14,25,29,30,32]. Our phylogenetic analysis of coexistence dynamics is at least partially consistent with this body of work by identifying the critical importance of evolutionary time in enabling the accumulation of sympatry following the generation of species in allopatry. However, our results also reveal that these historical effects are not sufficient on their own to explain patterns of coexistence and that the formation of species richness gradients thus depends on how energy availability determines the assembly of species into communities.

While resolving the historical dynamics of coexistence based on current species distributions is challenging, our analyses suggest that energy availability has relatively little influence on the rate at which coexistence is established following speciation, and that the predominant effect of energy availability is to maintain coexistence over longer periods of time. This effect of productivity on the duration of coexistence suggests that the key factor is not an accelerated transition into sympatry as a result of weaker or more diffuse species interactions or by faster rates of character displacement [61,75]. Indeed, it has previously been shown that negative species interactions can constrain the establishment of coexistence following speciation in vertebrates, even in highly productive tropical regions [47,48,54]. Instead, our results are consistent with the theory that higher energy availability, acting either directly or indirectly on population dynamics [18] and niche partitioning [24], reduces the rate of local extinction, ultimately allowing more species to be “packed” into productive tropical ecosystems [23].

The relative importance of ecological mechanisms linking energy availability to coexistence remains to be resolved. In particular, while the total biomass and numerical abundance of avian communities appears to generally increase with ecosystem productivity, implying a reduction in local extinction, whether this can account for the magnitude of observed differences in coexistence is unclear [3,36,76]. The increased vegetation complexity supported by higher energy environments seems a prime candidate for facilitating the extended coexistence of ecologically similar bird species [24,77], whereas other energy-related processes may exert a similar influence by facilitating local adaptation [78]. It also seems likely that processes enhancing coexistence will interact synergistically with macroevolutionary diversification, the main alternative explanation for the accumulation of higher species richness in productive regions [14,25,29,32]. Indeed, according to models of adaptive radiation, a greater capacity for local coexistence is expected to elevate both rates of diversification and species-carrying capacities at the regional scale [1,46].

Ultimately, how greater fluxes of energy and the concomitant increases in resource availability influence species coexistence will likely depend on context, region, scale, and clade. Nevertheless, our results suggest that energetic constraints on coexistence play a fundamental role in shaping contemporary gradients in species richness, and form a vital component of any mechanistic explanation for global patterns in biodiversity.

## Materials and Methods

### Sister Species Ages and Coexistence

Avian sister pairs and their estimated divergence times (Ma) were extracted from the Jetz et al. [14] time-calibrated phylogeny, pruned to contain only those species represented by genetic data (*n* = 6,670) and based on the primary backbone topology proposed by Hackett et al. [79]. To account for phylogenetic uncertainty in sister pairings and divergence times, we repeated our analysis across 100 trees drawn at random from the posterior distribution. All reported values and results represent the median across trees (trees can be downloaded from http://birdtree.org). We also conducted our analysis across a single maximum clade credibility (MCC) tree generated using TREEANNOTATOR (included in BEAST v.1.6.1) [80]. Results based on the MCC tree were highly concordant with our main analysis and are presented in S2 Table.

From our dataset of sister species pairs *(n* = 1,817), we excluded (i) very recently diverged species (<0.75 Ma, *n* = 236) in which ongoing introgression and ancestral polymorphism may confound reliable estimates of splitting events [81]; and (ii) species from poorly sampled genera (sampling <70%, *n* = 638) in which pairs are unlikely to represent true sister species. For the remaining sister species, we quantified coexistence by combining information on overlap of species breeding distributions and broad-scale habitat occupancy. For each sister pair, we estimated the area of distributional overlap from rasterised (1 km resolution) expert opinion maps of extent of occurrence (available to view at http://mol.org) [82]. We quantified percentage of range overlap between species according to the Szymkiewicz-Simpson coefficient [Area_Overlap_/min(Area_Sister1_, Area_Sister2_)] [61,83,84]. Sisters with abutting distributions or overlapping only marginally along narrow contact zones do not represent true coexistence and are thus sensitive to errors of commission, which increase strongly with spatial map resolution below ca. 150 km grain [85]. To ensure that our results are robust to these mapping errors, we repeated analyses using a range of different overlap thresholds (5%, 20%, 50%, 80%) to define coexistence. Unless otherwise stated, results presented in the main text refer to those based on the 20% overlap threshold commonly used in studies of species sympatry [61,83,84].

Species with overlapping (i.e., sympatric) breeding distributions may occupy the same (syntopic) or different (allotopic) habitats, but only where species are syntopic is energy availability expected to constrain coexistence [86]. We therefore used information on species altitudinal and habitat preferences to identify sister species pairs occupying distinct major habitat types or elevation zones *(n* = 127). Sister species occupying nonoverlapping elevation zones (in accordance with polygon range data defined as <20% overlap in elevation range) were identified using data on minimum and maximum elevation ranges compiled from a variety of published sources subjected to thorough cross-checking and updated to match current taxonomy [87–89]. Habitats were classified as forest, shrubland, bare ground, wetland, and “other” based on published information [89]. Finally, we excluded species pairs for which estimates of energy availability were either unavailable in areas of sympatry or unlikely to represent foraging areas (e.g., species breeding on islands but predominantly foraging at sea). In total, *n* = 1,021 sister pairs were included in our main analysis.

To map and test the environmental predictors of coexistence, we extracted polygon range maps onto an equal area grid (resolution of 110 km ≈ 1° at the equator) [82]. The incidence of coexistence was mapped as the proportion of sister species pairs coexisting within each grid cell. Because sister species often coexist over only part of their geographic range, we ensured that species pairs only contribute to positive cases of coexistence where they both occur; that is, cells occupied by only a single sister contribute a value of zero to the incidence of coexistence in those cells even if those species coexist in other areas, whereas cells occupied by both species contribute a value of one. Sister species age was mapped as the median age across pairs present within each cell. Maps in the main text show the median cell values from across the posterior distribution of trees.

### Environmental and Intrinsic Predictors of Coexistence

We used the same equal area grid for extracting species distributions to sample environmental and geographical data for each sister pair, focusing on two standard global layers representing alternative metrics of energy availability [90]. First, we used consensus estimates from a large model intercomparison (Fig 1B, [91]) to estimate mean annual energy available to heterotrophs or NPP (gCM^-2^, 30′ resolution, reflected and square-root-transformed). Annual, rather than seasonal, estimates were used because, in addition to the direct effects of resource abundance on individual growth rates and population density during the breeding season, productivity is expected to influence coexistence year-round through its effects on vegetation structural complexity and resource variety [24]. Second, we used the layers of Ahn and Tateishi [92] as estimates of global variation in actual evapotranspiration.

To account for possible covariance with other environmental factors, we assessed a number of putative predictors of species coexistence: mean annual temperature [57] (temperature: data from 1961 to 1990 with 10′ original resolution, reflected and LN-transformed) [93]; temperature and precipitation seasonality [2,94] (calculated as the average three-month intra-annual variance, based on the same sources as in Jetz and Rubenstein [95], LN-transformed); topographic heterogeneity [56] (elevation range: GTOPO30 (USGS 1996) range in elevation with 30′ resolution, square-root-transformed); and the difference in mean annual temperature between the Last Glacial Maximum (LGM; 21 kya) and the present day (LGM temperature anomaly, an index of long-term climatic variability, LN-transformed) [54,55]. Estimates of past climate were obtained from the Paleoclimate Modelling Intercomparison Project Phase II (MIROC3.2 coupled ocean–atmosphere model, originally in 2.5′ resolution) [96]. Although estimates of climatic variability would ideally be integrated over time, we note that the median age of sister species pairs in our dataset is 3.81 Ma, and, thus, the LGM temperature anomaly is likely to represent the spatial patterns of climatic variability over timescales relevant to our analysis (see [97]).

To determine the position of species pairs across the global gradient in each environmental variable, we calculated the mean conditions across the combined geographic range of both species (for allopatric sister pairs) or those cells where both species were present (for sympatric sister pairs). Coexistence between species is generally restricted to particular spatial locations, and by only calculating conditions in areas where both species are present, we were able to directly match the incidence of coexistence to the local environment (S3 Fig). Sister species pairs were assigned the biogeographic realm containing the majority of their combined geographic range.

We quantified relative dispersal ability using the hand-wing index (HWI) [51,98], a measure of the wing aspect-ratio that is a strong determinant of long-distance flight efficiency and both natal and migratory dispersal distances [64,99]. Following Claramunt [98], the HWI was calculated as

$$HWI=\frac{100\times Kipp’s\;distance}{wing\;chord}$$

where wing chord is the distance from the carpal joint (wrist) to the tip of the longest primary, and Kipp’s distance is the distance between the tips of the longest primary feather and the first secondary feather, both measured on the closed wing. Measurements were obtained from museum specimens, with a mean of five individuals per species (we aimed for a minimum of two individuals of each sex). We excluded two sister pairs for which wing data were unavailable and used the average HWI of each sister pair (square-root-transformed) in our analysis. Kipp’s distances for flightless species of the genus Apteryx, which retain only a vestigial wing, could not be measured, and so these species were assigned the minimum HWI observed across the dataset.

### The Incidence and Temporal Dynamics of Coexistence

We examined the relationship between species coexistence and energy availability using two different modelling frameworks. First, we treated coexistence as a binary trait and tested its predictors in a generalised linear model with a binomial error structure. To account for the possible effects of other variables that may co-vary with energy availability, we fitted multipredictor models including each environmental variable as a main effect. We included quadratic terms to account for potential nonlinearity in the relationship between each variable and coexistence probability. To allow comparison among effect sizes, we normalised variables to unit variance. Temperature seasonality was highly collinear with energy availability (Pearson’s r = −0.83), and so we excluded this variable from our model. We note that results remained qualitatively unchanged when including temperature seasonality (S3 and S4 Tables). In a further analysis, we focused only on coexisting species sisters (*n* = 187) and used a paired design to compare mean environmental conditions in zones of allopatry (i.e., nonoverlap) and coexistence (i.e., overlap).

If coexistence is determined by phylogenetically inherited traits, then treating sister pairs as independent may overestimate the significance of any association between the incidence of coexistence and local environment conditions. To evaluate this possibility, we calculated the phylogenetic signal in coexistence using the D statistic in the R package Caper [53,100]. This metric compares the distribution of a binary trait (1, 0) across the tips of the tree to two null models: (i) a Brownian motion model of trait evolution and (ii) a random trait distribution generated by shuffling species tip values. A value of D = 1 indicates a random distribution, while a value of D = 0 is the expectation under Brownian motion. The significance of the departure of the observed patterns from these two expectations is assessed through simulation *(n* = 1,000). Values of D can also extend beyond the range of 0–1. In these cases, D > 1 indicates greater overdispersion compared to a phylogenetically random pattern, while D < 0 indicates greater conservatism than expected under Brownian motion [53].

We found that the phylogenetic signal in coexistence is low but detectable (D = 0.88). Thus, to ensure that our results were robust to phylogenetic nonindependence, we repeated our analysis using phylogenetic mixed models fitted using Bayesian Markov Chain Monte Carlo methods in the R package MCMCglmm [101]. We included the phylogenetic covariance between species pairs as a random effect and a probit link function. Because MCMCglmm assumes an ultrametric tree, we modelled covariance among sister pairs using the evolutionary distances at the present day rather than the time at which sister species diverged. However, this is unlikely to influence our results, because the median age of sister pairs (3.81 Ma) is young compared to the age of the tree (98 Ma). We ran all models for 1 million iterations with a burn-in of 50,000 iterations and a thinning interval of 100 iterations. We set flat noninformative priors with a low degree of belief across all variables.

Second, we modelled the dynamics of species coexistence over time as a constant-rate Markov process and examined the effects of energy availability on both the waiting time to coexistence and the duration of coexistence. In this model, we assumed that at the time of population divergence, sister species have allopatric distributions [46,50,102]. Given the observed time since divergence (Ma) and the current geographical relationship of each sister pair (allopatric or coexisting), we then used maximum likelihood to estimate the per-lineage rate of transition from allopatry to coexistence (σ) and the reverse transition back to allopatry (ε) [47,51]. Using this approach, we compared the fit of a null model in which transition rates were equivalent across species (n_parameters_ = 2) to an “energy-availability dependent” (EAD) model in which NPP was included as a covariate on either σ or ε (n_parameters_ = 3). Finally, we fitted an EAD model in which NPP had independent effects on both σ and ε (n_parameters_ = 4). In each case, we tested for an improvement in model fit using AIC. We assessed the relative ability of these models to accurately explain patterns of coexistence by using our model parameter estimates to predict the incidence of coexistence as a continuous function of both species age and local productivity. Models were fitted in the R package msm [103]. Simulation tests demonstrating that σ and ε can be reliably inferred given present day information on sister species coexistence and divergence times are described in S1 Text and S4 Fig.

## Supporting Information

S1 Fig The distribution of extant sister species pairs across the avian evolutionary tree, highlighting pairs that are currently in geographic isolation (blue) or coexistence (red). Results are shown for a single tree from the posterior distribution and including only species containing genetic data (6,670 species). Our analyses were run over a total of 100 trees. N = number of pairs.
(TIF)

S2 Fig Geographical variation in sister species richness across grid cells (*n* = 10,938 cells). (A) The relationship between the richness of all sister species in the analysis (*n* = 1,021 x 2 species) and the total richness of all bird species (*n* = 9,993 species). (B) The relationship between the richness of locally coexisting sister species and the richness of all sister species (*n* = 1,021 x 2 species). In (B), the dashed line indicates a 1:1 relationship. Points falling along this line would indicate that all sister species in a grid cell are members of locally coexisting pairs. (A) Sister species richness strongly co-varies with total avian richness. (B) Cells containing many coexisting sisters support more sister species pairs overall. See Dryad depository for cell values [52].
(TIF)

S3 Fig Examples of geographic isolation (allopatry; unfilled squares) and coexistence (sympatry; grey diagonal lines) between sister species of African Bee-eaters (*Merops*). In (A), sister species are completely spatially segregated; in (B), sister species coexist but are allopatric in some parts of their range. Insets show the distribution of NPP values across areas of geographic isolation (blue) and coexistence (red) for each pair. Heat-map denotes NPP (gCM^-2^), with hotter colours indicating cells with higher productivity. Sister species are (A) *Merops bullockoides* (solid white outline) and *Merops bulocki* (solid black outline), and (B) *Merops gularis* (solid white outline) and *Merops muelleri* (solid black outline).
(TIF)

S4 Fig Reliability of estimates of the transition rate from allopatry to sympatry (σ, top panel) and from sympatry to allopatry (ε, bottom panel) for different species ages (mean age = 1.5, 3.3, and 10 Ma). Colours denote the values of σ and ε used in the simulation. Box plots show the spread of estimated values from 100 replicate simulations and lines the true (i.e., simulated) value. See Dryad depository for simulated data [52].
(TIF)

S1 Table Predictors of species coexistence across pairs (*n* = 1,021 pairs) and within pairs (*n* = 187 pairs) for the posterior distribution of trees. Results are shown for both univariate and multivariate models and for all four range overlap thresholds (5%, 20%, 50%, 80%) used to define coexistence.
(DOCX)

S2 Table Predictors of species coexistence across pairs (*n* = 1,028 pairs) for the maximum clade credibility (MCC) tree. Results are shown for both univariate and multivariate models and for all four range overlap thresholds (5%, 20%, 50%, 80%) used to define coexistence.
(DOCX)

S3 Table Predictors of species coexistence across pairs (*n* = 1,021 pairs) for models including both NPP and temperature seasonality. Results are shown for both univariate and multivariate models and for all four range overlap thresholds (5%, 20%, 50%, 80%) used to define coexistence.
(DOCX)

S4 Table Multipredictor phylogenetic mixed models of species coexistence (*n* = 1,021 pairs) fitted across the posterior distribution of trees. (DOCX)

S5 Table Model parameter estimates for the Null and energy-availability dependent (EAD) model of coexistence dynamics *(n* = 1,021 pairs). Hazard ratios indicate the relative change in the transition rate to coexistence (σ) and segregation (ε) between minimum and maximum NPP.
(DOCX)

S6 Table Model parameter estimates for the Null and latitudinal dependent (LD) model of coexistence dynamics fit to all (“Global;” *n* = 1,021 pairs) and New World (*n* = 492 pairs) sisters. Hazard ratios indicate the relative change in the transition rate to coexistence (σ) and segregation (ε) between minimum and maximum absolute geographic latitude.
(DOCX)

S7 Table The relationship between total avian species richness *(n* = 9,993 species) and sister species coexistence (percentage; *n* = 1,021 pairs) accounting for biogeographic realm (*n* = 8,471 cells). Effect sizes show contrasts to the Australian realm.
(DOCX)

S1 Text Simulation tests of model reliability and precision. (DOCX)

## Abbreviations

AIC: Akaike Information Criterion
AET: actual evapotranspiration
EAD: energy-availability dependent
GLM: generalised linear model
HWI: hand-wing index
LD: latitudinal dependent
Ma: million years
NPP: net primary productivity
