# Supplementary figures and images for: Energetic Constraints on Species Coexistence in Birds
Source: PLoS Biol. 2016 Mar 14;14(3):e1002407. doi: 10.1371/journal.pbio.1002407 (PMC4790906; doi:10.1371/journal.pbio.1002407)

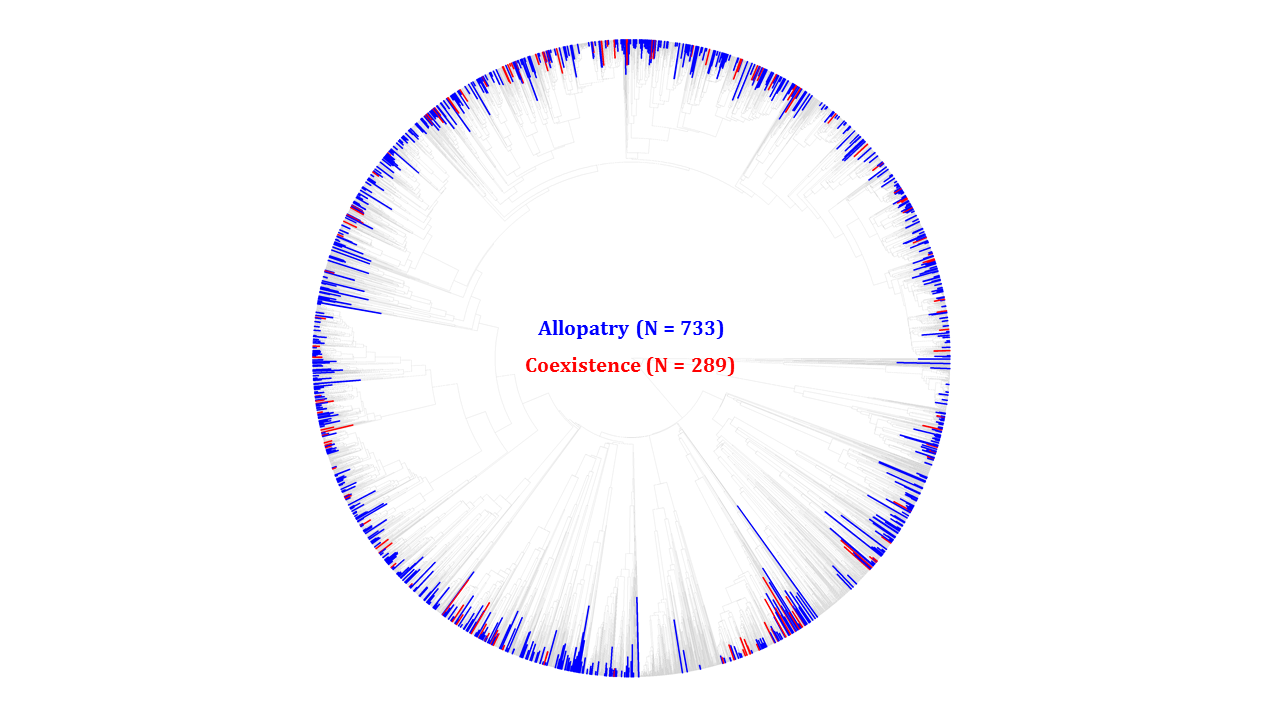

Supplement: S1 Fig — Results are shown for a single tree from the posterior distribution and including only species containing genetic data (6,670 species). Our analyses were run over a total of 100 trees. N = number of pairs. (TIF) [file pbio.1002407.s001.tif]

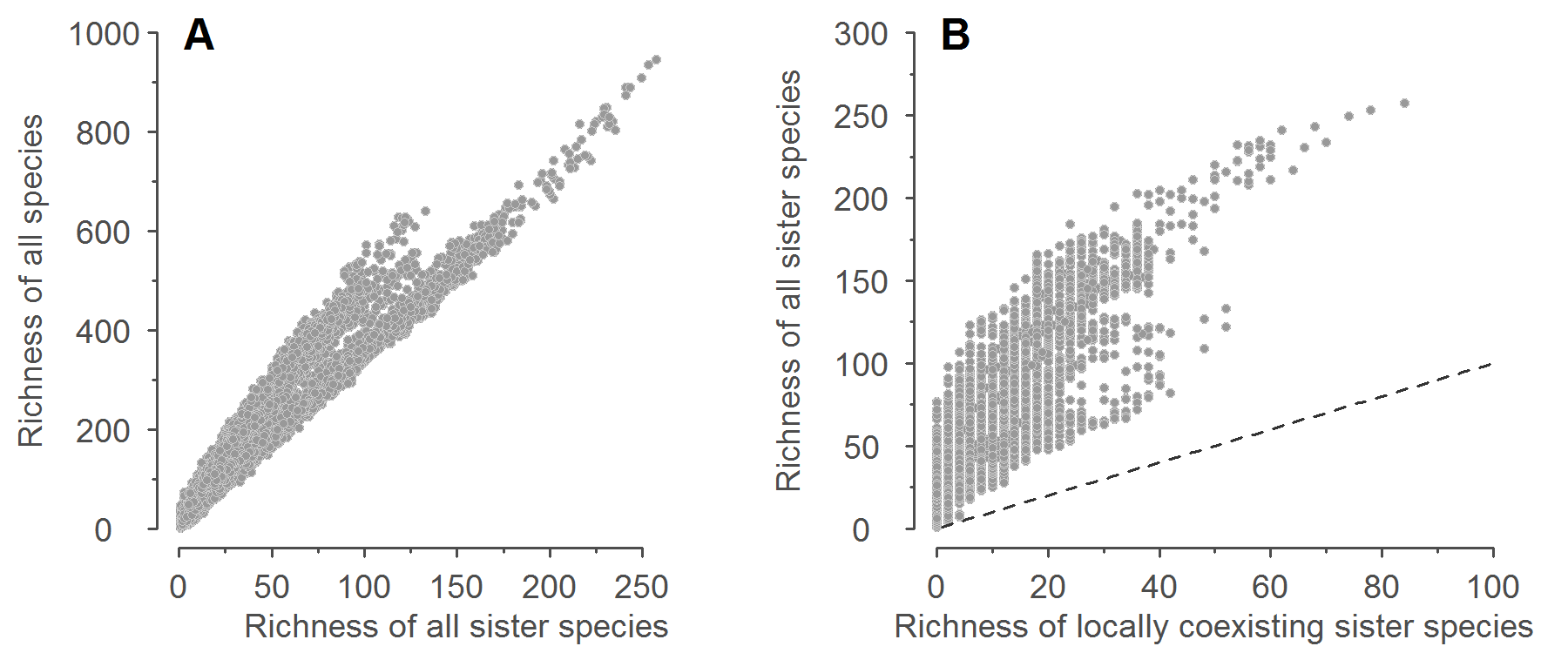

Supplement: S2 Fig — (A) The relationship between the richness of all sister species in the analysis (n = 1,021 x 2 species) and the total richness of all bird species (n = 9,993 species). (B) The relationship between the richness of locally coexisting sister species and the richness of all sister species (n = 1,021 x 2 species). In (B), the dashed line indicates a 1:1 relationship. Points falling along this line would indicate that all sister species in a grid cell are members of locally coexisting pairs. (A) Sister species richness strongly co-varies with total avian richness. (B) Cells containing many coexisting sisters support more sister species pairs overall. See Dryad depository for cell values [52]. (TIF) [file pbio.1002407.s002.tif]

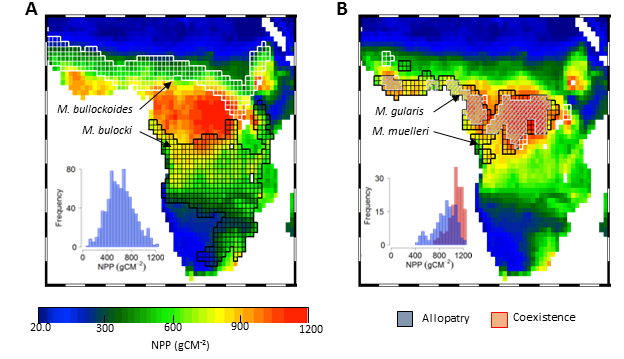

Supplement: S3 Fig — In (A), sister species are completely spatially segregated; in (B), sister species coexist but are allopatric in some parts of their range. Insets show the distribution of NPP values across areas of geographic isolation (blue) and coexistence (red) for each pair. Heat-map denotes NPP (gCM-2), with hotter colours indicating cells with higher productivity. Sister species are (A) Merops bullockoides (solid white outline) and Merops bulocki (solid black outline), and (B) Merops gularis (solid white outline) and Merops muelleri (solid black outline). (TIF) [file pbio.1002407.s003.tif]

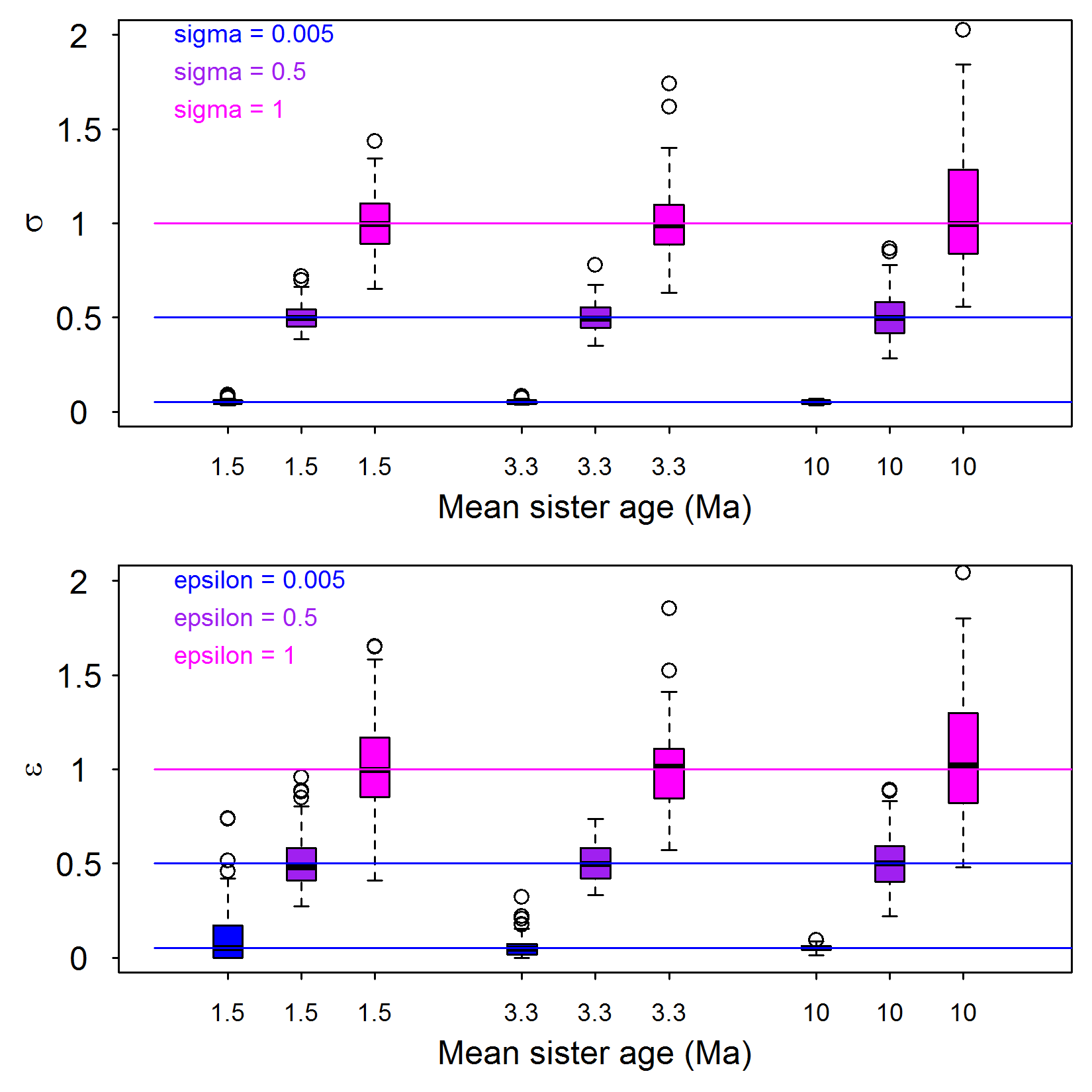

Supplement: S4 Fig — Colours denote the values of σ and ε used in the simulation. Box plots show the spread of estimated values from 100 replicate simulations and lines the true (i.e., simulated) value. See Dryad depository for simulated data [52]. (TIF) [file pbio.1002407.s004.tif]
