# Supplementary material for: Energetic Constraints on Species Coexistence in Birds
Source: PLoS Biol. 2016 Mar 14;14(3):e1002407. doi: 10.1371/journal.pbio.1002407 (PMC4790906; doi:10.1371/journal.pbio.1002407)
Supplement: S1 Table — Results are shown for both univariate and multivariate models and for all four range overlap thresholds (5%, 20%, 50%, 80%) used to define coexistence. (DOCX) [file pbio.1002407.s005.docx]

|  | Across pair | | | | | | | | | | | | |  | Within pair | |
| --- | --- | --- | --- | --- | --- | --- | --- | --- | --- | --- | --- | --- | --- | --- | --- | --- |
|  | Univariate | | | | | | | |  | Multivariate | | | |  | Univariate | Multivariate |
|  | 5% | | 20% | | 50% | | 80% | |  | 5% | 20% | 50% | 80% |  | 20% | 20% |
|  | β | AIC | β | AIC | β | AIC | β | AIC |  | β | β | β | β |  | β | β |
| NPP | 0.166** | 1354.9 | 0.208** | 1201.8 | 0.226** | 990.6 | 0.392*** | 679.8 |  | 0.427** | 0.409* | 0.497** | 0.652** |  | 0.308** | 0.976** |
| NPP^2^ | 0.17** |  | 0.211** |  | 0.15* |  | 0.167 |  |  | 0.189** | 0.195* | 0.114 | 0.135 |  | 0.33** | 0.36** |
| Precipitation seasonality | -0.132* | 1361.8 | -0.263*** | 1205.9 | -0.269*** | 991.1 | -0.374*** | 687.0 |  | 0.148 | 0.02 | 0.065 | 0.102 |  | -0.365** | 0.074 |
| Elevation range | -0.154* | 1335.6 | -0.034 | 1168.6 | -0.183* | 974.5 | -0.268** | 690.7 |  | -0.211* | -0.078 | -0.269* | -0.316* |  | -0.137 | -0.37* |
| Elevation range^2^ | 0.338*** |  | 0.421*** |  | 0.365*** |  | 0.268*** |  |  | 0.345*** | 0.44*** | 0.388*** | 0.316*** |  | 0.447*** | 0.513*** |
| Temperature | 0.058 | 1365.7 | 0.004 | 1219.5 | 0.061 | 1001.4 | 0.275* | 693.6 |  | -0.269 | -0.285 | -0.369 | -0.221 |  | -0.096 | -0.784** |
| LGM temperature anomaly | -0.253* | 1364.7 | -0.411*** | 1209.8 | -0.484*** | 989.3 | -0.586*** | 690.6 |  | -0.182 | -0.357* | -0.412* | -0.401 |  | 0.017 | -0.087 |
| LGM temperature anomaly^2^ | 0.087* |  | 0.147** |  | 0.181*** |  | 0.206*** |  |  | 0.074 | 0.136** | 0.157** | 0.195** |  | 0.01 | 0.005 |
| HWI | 0.078 | 1365.6 | 0.055 | 1218.6 | -0.044 | 1001.8 | -0.146 | 697.9 |  | 0.113 | 0.107 | 0.000 | -0.068 |  |  |  |
| Age | 0.521*** | 1346.5 | 0.588*** | 1196.1 | 0.657*** | 978.5 | 0.783*** | 682.0 |  | 0.521*** | 0.615*** | 0.655*** | 0.755*** |  |  |  |

Numbers are z-values; ^2^ denotes quadratic effect; AIC is Akaike Information Criterion; stars represent significance levels at *P* < 0.05 (*), 0.01 (**), 0.001 (***). Values are the median across N = 100 trees.
