# Supplementary material for: Energetic Constraints on Species Coexistence in Birds
Source: PLoS Biol. 2016 Mar 14;14(3):e1002407. doi: 10.1371/journal.pbio.1002407 (PMC4790906; doi:10.1371/journal.pbio.1002407)
Supplement: S2 Table — Results are shown for both univariate and multivariate models and for all four range overlap thresholds (5%, 20%, 50%, 80%) used to define coexistence. (DOCX) [file pbio.1002407.s006.docx]

|  | Univariate | | | | | | | |  | Multivariate | | | |
| --- | --- | --- | --- | --- | --- | --- | --- | --- | --- | --- | --- | --- | --- |
|  | 5% | | 20% | | 50% | | 80% | |  | 5% | 20% | 50% | 80% |
|  | β | AIC | β | AIC | β | AIC | β | AIC |  | β | β | β | β |
| NPP | 0.189** | 1361.9 | 0.241*** | 1218.1 | 0.238** | 1009.5 | 0.386*** | 681.3 |  | 0.43** | 0.41* | 0.414* | 0.613** |
| NPP^2^ | 0.183** |  | 0.206** |  | 0.121 |  | 0.156 |  |  | 0.196** | 0.186* | 0.064 | 0.095 |
| Precipitation seasonality | -0.128* | 1372.4 | -0.254*** | 1224.9 | -0.263*** | 1009 | -0.356*** | 687.1 |  | 0.145 | 0.017 | -0.019 | 0.075 |
| Elevation range | -0.197** | 1342.3 | -0.084 | 1187.5 | -0.264*** | 983.86 | -0.352*** | 680.6 |  | -0.234** | -0.103 | -0.3** | -0.354** |
| Elevation range^2^ | 0.347*** |  | 0.425*** |  | 0.4*** |  | 0.319*** |  |  | 0.356*** | 0.444*** | 0.424*** | 0.369*** |
| Temperature | 0.092 | 1374.2 | 0.059 | 1237.5 | 0.11 | 1018 | 0.292** | 691.5 |  | -0.234 | -0.233 | -0.248 | -0.125 |
| LGM temperature anomaly | -0.311** | 1371.0 | -0.465*** | 1225.6 | -0.499*** | 1007.4 | -0.525** | 690.0 |  | -0.239 | -0.407** | -0.4* | -0.296 |
| LGM temperature anomaly^2^ | 0.104* |  | 0.158*** |  | 0.185*** |  | 0.202*** |  |  | 0.089 | 0.151** | 0.152** | 0.181* |
| HWI | 0.114 | 1373.3 | 0.088 | 1236.7 | 0.026 | 1019.8 | -0.112 | 698.1 |  | 0.165* | 0.154* | 0.07 | -0.057 |
| Age | 0.58*** | 1347.7 | 0.607*** | 1210.9 | 0.631*** | 997.2 | 0.722*** | 681.0 |  | 0.566*** | 0.619*** | 0.632*** | 0.698*** |

β are slope estimates; ^2^ denotes quadratic effect; AIC is Akaike Information Criterion; stars represent significance levels at *P* < 0.05 (*), 0.01 (**), 0.001 (***).
