# Supplementary material for: Energetic Constraints on Species Coexistence in Birds
Source: PLoS Biol. 2016 Mar 14;14(3):e1002407. doi: 10.1371/journal.pbio.1002407 (PMC4790906; doi:10.1371/journal.pbio.1002407)
Supplement: S3 Table — Results are shown for both univariate and multivariate models and for all four range overlap thresholds (5%, 20%, 50%, 80%) used to define coexistence. (DOCX) [file pbio.1002407.s007.docx]

|  | Across pair | | | | | | | | | | | | |
| --- | --- | --- | --- | --- | --- | --- | --- | --- | --- | --- | --- | --- | --- |
|  | Univariate | | | | | | | |  | Multivariate | | | |
|  | 5% | | 20% | | 50% | | 80% | |  | 5% | 20% | 50% | 80% |
|  | β | AIC | β | AIC | β | AIC | β | AIC |  | β | β | β | β |
| NPP | 0.166** | 1354.9 | 0.208** | 1201.8 | 0.226** | 990.6 | 0.392*** | 679.8 |  | 0.32 | 0.378 | 0.522* | 0.629* |
| NPP^2^ | 0.17** |  | 0.211** |  | 0.15* |  | 0.167 |  |  | 0.178* | 0.194* | 0.118 | 0.134 |
| Precipitation seasonality | -0.132* | 1361.8 | -0.263*** | 1205.9 | -0.269*** | 991.1 | -0.374*** | 687.0 |  | 0.149 | 0.022 | 0.065 | 0.101 |
| Temperature seasonality | -0.197** | 1357.6 | -0.246*** | 1207.9 | -0.209** | 995.0 | -0.368*** | 688.6 |  | -0.239 | -0.05 | 0.055 | -0.019 |
| Elevation range | -0.154* | 1335.6 | -0.034 | 1168.6 | -0.183* | 974.5 | -0.268** | 690.7 |  | -0.269** | -0.094 | -0.249* | -0.326* |
| Elevation range^2^ | 0.338*** |  | 0.421*** |  | 0.365*** |  | 0.268*** |  |  | 0.317*** | 0.433*** | 0.396*** | 0.315** |
| Temperature | 0.058 | 1365.7 | 0.004 | 1219.5 | 0.061 | 1001.4 | 0.275* | 693.6 |  | -0.39* | -0.319 | -0.338 | -0.239 |
| LGM temperature anomaly | -0.253* | 1364.7 | -0.411*** | 1209.8 | -0.484*** | 989.3 | -0.586*** | 690.6 |  | -0.164 | -0.353* | -0.414* | -0.402 |
| LGM temperature anomaly^2^ | 0.087* |  | 0.147** |  | 0.181*** |  | 0.206*** |  |  | 0.069 | 0.136* | 0.157** | 0.194** |
| HWI | 0.078 | 1365.6 | 0.055 | 1218.6 | -0.044 | 1001.8 | -0.146 | 697.9 |  | 0.108 | 0.104 | 0.002 | -0.071 |
| Age | 0.521*** | 1346.5 | 0.588*** | 1196.1 | 0.657*** | 978.5 | 0.783*** | 682.0 |  | 0.529*** | 0.619*** | 0.655*** | 0.755*** |

β are slope estimates; ^2^ denotes quadratic effect; AIC is Akaike Information Criterion; stars represent significance levels at *P* < 0.05 (*), 0.01 (**), 0.001 (***). Multivariate model includes both NPP and temperature seasonality. Values are the median across N = 100 trees.
