# Supplementary material for: Energetic Constraints on Species Coexistence in Birds
Source: PLoS Biol. 2016 Mar 14;14(3):e1002407. doi: 10.1371/journal.pbio.1002407 (PMC4790906; doi:10.1371/journal.pbio.1002407)
Supplement: S4 Table — (DOCX) [file pbio.1002407.s008.docx]

| Predictors | β1 |  | β2 |  |
| --- | --- | --- | --- | --- |
| NPP | 0.360 | * | 0.360 | * |
| NPP^2^ | 0.250 | ** | 0.171 | * |
| Precipitation seasonality | -0.064 |  | 0.006 |  |
| Precipitation seasonality^2^ | -0.029 |  | -0.021 |  |
| Temperature seasonality | -0.082 |  |  |  |
| Temperature seasonality^2^ | -0.271 | * |  |  |
| Elevation range | -0.034 |  | -0.030 |  |
| Elevation range ^2^ | 0.389 | *** | 0.393 | *** |
| Temperature | -0.308 |  | -0.230 |  |
| Temperature^2^ | 0.169 | * | 0.087 |  |
| LGM temperature anomaly^2^ | -0.366 | * | -0.438 | ** |
| LGM temperature anomaly ^2^ | 0.126 | * | 0.128 | ** |
| HWI | 0.231 | * | 0.219 | * |
| HWI^2^ | -0.062 |  | -0.059 |  |
| Age | 0.569 | *** | 0.568 | *** |
| Age^2^ | -0.039 |  | -0.029 |  |
| DIC | 1037.58 |  | 1039.44 |  |

β are slope estimates; ^2^ denotes quadratic effect; DIC is Deviance Information Criterion; stars represent significance levels at *P* < 0.05 (*), 0.01 (**), 0.001 (***). β1: model including temperature seasonality; β2: model excluding temperature seasonality. Values are the median across N = 100 trees.

n.
