# Supplementary material for: Energetic Constraints on Species Coexistence in Birds
Source: PLoS Biol. 2016 Mar 14;14(3):e1002407. doi: 10.1371/journal.pbio.1002407 (PMC4790906; doi:10.1371/journal.pbio.1002407)
Supplement: S5 Table — Hazard ratios indicate the relative change in the transition rate to coexistence (σ) and segregation (ε) between minimum and maximum NPP. (DOCX) [file pbio.1002407.s009.docx]

| Model | Transitions | σ | ε | σ Hazard  ratio | ε Hazard ratio | AIC | ∆AIC |
| --- | --- | --- | --- | --- | --- | --- | --- |
| Null | 1-way | 0.07 ± (0.06:0.08) |  |  |  | 1267.54 |  |
| Null | 2-way | 0.17 ± (0.12:0.24) | 0.3 ± (0.17:0.52) |  |  | 1199.49 |  |
| EAD (σ) | 1-way | 0.07 ± (0.06:0.08) |  | 1.74 ± (1.07:2.81) |  | 1264.37 | 2.96 |
| EAD (ε) | 2-way | 0.18 ± (0.13:0.25) | 0.32 ± (0.19:0.55) |  | 0.28 ± (0.13:0.63) | 1191.87 | 7.92 |
| EAD (σ) | 2-way | 0.17 ± (0.12:0.25) | 0.31 ± (0.17:0.55) | 2.81 ± (1.25:3.81) |  | 1192.93 | 5.72 |
| EAD (σ + ε) | 2-way | 0.18 ± (0.12:0.25) | 0.32 ± (0.18:0.56) | 1.07 ± (0.37:3.32) | 0.32 ± (0.07:1.5) | 1193.60 | 6.05 |

Model: Denotes whether transition rate to coexistence (σ), segregation (ε), both (σ + ε) are dependent (EAD) or invariant (Null) with energy availability. Transitions: Denotes whether the reverse transition rate from coexistence to segregation was fixed at zero (“1-way”) or estimated (“2-way”) from the data. Hazard ratio: Hazard ratios indicate the relative change in the transition rate to coexistence (σ) and segregation (ε) between minimum and maximum NPP. A value >1 indicates an increase in transition rate with energy availability, while a value <1 indicates that the specified transition rate decreases with energy availability. AIC is Akaike Information Criterion; ∆AIC is the difference in AIC between the Null and EAD model. Values are the median across N = 100 trees.
