# Supplementary material for: Energetic Constraints on Species Coexistence in Birds
Source: PLoS Biol. 2016 Mar 14;14(3):e1002407. doi: 10.1371/journal.pbio.1002407 (PMC4790906; doi:10.1371/journal.pbio.1002407)
Supplement: S6 Table — Hazard ratios indicate the relative change in the transition rate to coexistence (σ) and segregation (ε) between minimum and maximum absolute geographic latitude. (DOCX) [file pbio.1002407.s010.docx]

|  | Model | Transitions | σ | ε | σ Hazard ratio | ε Hazard ratio | AIC | ∆AIC |
| --- | --- | --- | --- | --- | --- | --- | --- | --- |
| Global | Null | 1-way | 0.07 ± (0.06:0.08) |  |  |  | 1267.54 |  |
| Global | Null | 2-way | 0.17 ± (0.12:0.24) | 0.3 ± (0.17:0.52) |  |  | 1199.49 |  |
| Global | LD (σ) | 1-way | 0.07 ± (0.06:0.08) |  | 1 ± (0.61:1.66) |  | 1269.32 | -1.91 |
| Global | LD (ε) | 2-way | 0.17 ± (0.12:0.24) | 0.3 ± (0.17:0.52) |  | 1.47 ± (0.63:3.55) | 1200.89 | -1.21 |
| Global | LD (σ) | 2-way | 0.17 ± (0.12:0.24) | 0.3 ± (0.17:0.53) | 0.84 ± (0.48:1.47) |  | 1201.30 | -1.63 |
| Global | LD (σ + ε) | 2-way | 0.17 ± (0.12:0.24) | 0.3 ± (0.17:0.52) | 1.37 ± (0.38:4.72) | 2.38 ± (0.31:15.67) | 1202.56 | -2.79 |
| New World | Null | 1-way | 0.08 ± (0.07:0.09) |  |  |  | 603.12 |  |
| New World | Null | 2-way | 0.17 ± (0.11:0.26) | 0.26 ± (0.12:0.58) |  |  | 573.55 |  |
| New World | LD (σ) | 1-way | 0.08 ± (0.06:0.09) |  | 2.15 ± (1.02:4.49) |  | 601.17 | 2.02 |
| New World | LD (ε) | 2-way | 0.18 ± (0.11:0.28) | 0.28 ± (0.12:0.65) |  | 0.45 ± (0.11:1.96) | 573.57 | -0.91 |
| New World | LD (σ) | 2-way | 0.16 ± (0.11:0.25) | 0.26 ± (0.12:0.56) | 2.39 ± (1.04:5.52) |  | 570.93 | 2.19 |
| New World | LD (σ + ε) | 2-way | 0.16 ± (0.1:0.24) | 0.23 ± (0.1:0.52) | 5.49 ± (1.2:25.01) | 6.02 ± (0.46:82.83) | 570.42 | 2.12 |

Model: Denotes whether transition rate to coexistence (σ), segregation (ε), both (σ + ε) are dependent (LD) or invariant (Null) with absolute geographic latitude. Transitions: Denotes whether the reverse transition rate from coexistence to segregation was fixed at zero (“1-way”) or estimated (“2-way”) from the data. Hazard ratio: Hazard ratios indicate the relative change in the transition rate to coexistence (σ) and segregation (ε) between minimum and maximum absolute latitude. A value >1 indicates an increase in transition rate with latitude while a value <1 indicates that the specified transition rate decreases with latitude. AIC = Akaike Information Criterion, ∆AIC is the difference in AIC between the Null and LD model. Values are the median across N = 100 trees.
