# Supplementary material for: Energetic Constraints on Species Coexistence in Birds
Source: PLoS Biol. 2016 Mar 14;14(3):e1002407. doi: 10.1371/journal.pbio.1002407 (PMC4790906; doi:10.1371/journal.pbio.1002407)
Supplement: S7 Table — Effect sizes show contrasts to the Australian realm. (DOCX) [file pbio.1002407.s011.docx]

|  | Effect | Standard error | *P* |
| --- | --- | --- | --- |
| Intercept | 4.67 | 0.04 | *** |
| Afrotropics | 0.75 | 0.04 | *** |
| Indomalaya | 0.76 | 0.04 | *** |
| Nearctic | 0.65 | 0.06 | *** |
| Neotropics | 0.47 | 0.05 | *** |
| Palearctic | 0.20 | 0.04 | *** |
| Coexistence (%) | 1.95 | 0.12 | *** |
| Afrotropics × Coexistence | -0.85 | 0.13 | *** |
| Indomalaya × Coexistence | -0.52 | 0.13 | *** |
| Nearctic × Coexistence | -2.27 | 0.16 | *** |
| Neotropics × Coexistence | 0.96 | 0.15 | *** |
| Palearctic × Coexistence | -0.80 | 0.13 | *** |

Effect size estimated from a generalised linear model (GLM) with quasi-Poisson error structure and are contrasts to the Australian realm. Stars represent significance levels at *P* < 0.05 (*), 0.01 (**), 0.001 (***). ‘Coexistence’ is the % of sister species coexisting per cell following angular transformation.
