# Supplementary material for: Energetic Constraints on Species Coexistence in Birds
Source: PLoS Biol. 2016 Mar 14;14(3):e1002407. doi: 10.1371/journal.pbio.1002407 (PMC4790906; doi:10.1371/journal.pbio.1002407)
Supplement: S1 Text — (DOCX) [file pbio.1002407.s012.docx]

**Simulation tests of model reliability and precision**

To confirm that our evolutionary transition model is able to reliably infer the historical dynamics of coexistence we conducted simulation tests. We generated a dataset sets of 1000 sister species pairs, following an exponential age distribution (mean age = 3.3 Ma). Assuming that sister species are initially in a state of allopatry, we then simulated coexistence dynamics along the branch length of each sister pair using known values of σ and ε (0.05, 0.5, 1). Following [[1](#_ENREF_1)], transitions between allopatry and coexistence occurred in continuous time according to a Gillespie algorithm [[2](#_ENREF_2)]. For each combination of parameters, we conducted 100 replicated simulations. We fitted our maximum likelihood model to each of these simulated datasets to assess how reliably we can estimate ε and σ. To demonstrate that estimated transitions rates, and thus the inferred waiting time to coexistence (1/σ) and duration of coexistence (1/ε), are not dependent on the observed gradient in sister species ages (Figure 1C), we repeated our analysis using a variety of sister species age distributions (mean age = 1.5, 3.3, 10 Ma).

The results show that our model can reliably estimate both σ and ε and that these estimates are robust to differences in species age (S4 Fig). As expected the precision of our estimates depend on the average age of sister species relative to the rates of coexistence dynamics (i.e. when species are either all very young or very old there is less information to estimate rates) but the estimates remain unbiased regardless of age (S4 Fig). These results confirm the suitability of our model for estimating historical coexistence dynamics given only the age and present day incidence of sympatry across sister species pairs.

References

1. Pigot AL, Tobias JA (2015) Dispersal and the transition to sympatry in vertebrates. Proceedings of the Royal Society B-Biological Sciences 282.

2. Gillespie DT (1977) Exact stochastic simulation of coupled chemical-reactions. Journal of Physical Chemistry 81: 2340-2361.

3. Hackett SJ, Kimball RT, Reddy S, Bowie RCK, Braun EL, et al. (2008) A phylogenomic study of birds reveals their evolutionary history. Science 320: 1763-1768.
